# Integrating nutrient bioavailability and co-production links when identifying sustainable diets: How low should we reduce meat consumption?

**DOI:** 10.1371/journal.pone.0191767

**Published:** 2018-02-14

**Authors:** Tangui Barré, Marlène Perignon, Rozenn Gazan, Florent Vieux, Valérie Micard, Marie-Josèphe Amiot, Nicole Darmon

**Affiliations:** 1 NORT, Aix-Marseille Univ, INSERM, INRA, Marseille, France; 2 MOISA, INRA, CIRAD, CIHEAM-IAMM, Montpellier SupAgro, Univ Montpellier, Montpellier, France; 3 MS-Nutrition, Faculté de Médecine de la Timone, Marseille, France; 4 IATE, Montpellier SupAgro, CIRAD, INRA, Univ Montpellier, Montpellier, France; McMaster University, CANADA

## Abstract

**Background:**

Reducing the consumption of meat and other animal-based products is widely advocated to improve the sustainability of diets in high-income countries. However, such reduction may impair nutritional adequacy, since the bioavailability of key nutrients is higher when they come from animal- vs plant-based foods. Meat reduction may also affect the balance between foods co-produced within the same animal production system.

**Objective:**

The objective was to assess the impact of introducing nutrient bioavailability and co-production links considerations on the dietary changes needed − especially regarding meat ‒ to improve diet sustainability.

**Methods:**

Diet optimization with linear and non-linear programming was used to design, for each gender, three modeled diets departing the least from the mean observed French diet (OBS) while reducing by at least 30% the diet-related environmental impacts (greenhouse gas emissions, eutrophication, acidification): i) in the nutrition-environment (NE) model, the fulfillment of recommended dietary allowances for all nutrients was imposed; ii) in the NE-bioavailability (NEB) model, nutritional adequacy was further ensured by accounting for iron, zinc, protein and provitamin A bioavailability; iii) in the NEB-co-production (NEB-CP) model, two links between co-produced animal foods (milk–beef and blood sausage–pork) were additionally included into the models by proportionally co-constraining their respective quantities. The price and environmental impacts of individual foods were assumed to be constant.

**Results:**

‘Fruit and vegetables’ and ‘Starches’ quantities increased in all modeled diets compared to OBS. In parallel, total meat and ruminant meat quantities decreased. Starting from 110g/d women’s OBS diet (168g/d for men), total meat quantity decreased by 78%, 67% and 32% for women (68%, 66% and 62% for men) in NE, NEB and NEB-CP diets, respectively. Starting from 36g/d women’s OBS diet (54g/d for men), ruminant meat quantity dropped severely by 84% and 87% in NE and NEB diets for women (80% and 78% for men), whereas it only decreased by 27% in NEB-CP diets (38% for men). The share of energy and proteins of animal origin was similar for the 3 modeled diets (approximately 1/5 of total energy, and 1/2 of protein) and lower than in OBS diet (approximately 1/3 of total energy, and 2/3 of protein).

**Conclusions:**

Decreasing meat content was strictly needed to achieve more sustainable diets for French adults, but the reduction was less severe when nutrient bioavailability and co-production links were taken into account.

## Introduction

The FAO defines sustainable diets as diets “with low environmental impacts […], protective and respectful of biodiversity and ecosystems, culturally acceptable, accessible, economically fair and affordable; nutritionally adequate, safe and healthy” [1]. Greenhouse gas emissions (GHGE) stemming from the agricultural sector amount to around 30% of global emissions, much of it coming from the livestock sector [2]. Global increase and intensification of animal and crop production also highly contribute to eutrophication and acidification [3–5]. Widespread adoption of plant-based diets has been identified as a potentially efficient way to reduce both the growing environmental burdens of global food consumption and the prevalence of diet-related chronic diseases [6,7]. However, this win-win situation between health and environment should also be weighed against other sustainability dimensions. Environmentally-friendlier, healthier diets may be less affordable [8,9], and plant-based diets may be less culturally acceptable [10] than currently consumed diets. Diet modeling using a mathematical approach such as linear (and non-linear) programming allows optimizing one function subjected to several constraints. It is therefore an ideal tool to simultaneously consider several dimensions of diet sustainability [11–14]. Applied to the French context, this approach showed that reducing dietary GHGE (down to -30%) while meeting nutritional recommendations was achievable through reduction of meat consumption and other moderate dietary shifts, without impairing the affordability of diet [12].

Recommended dietary allowances (RDA) set the daily intakes that meet the nutrient needs of 97.5% of the population. They are based on estimated nutrient requirement and average nutrient bioavailability, considering population’s usual dietary pattern [15]. However, nutrient bioavailability, defined as the proportion of an ingested nutrient absorbed and utilized through metabolic pathways, depends on host- (e.g. nutritional status) and diet-related factors [16], and may be lower for some nutrients when they are provided by plant- instead of animal-based foods [17]. Therefore, a shift toward more plant-based diets may not satisfy physiological requirements even if total ingested quantity is above the RDAs [18]. Several studies have assessed the environmental impact of actual and modeled diets to identify dietary changes that would help mitigating the environmental footprint of the food system [19,20]. However, to our knowledge, none of them took into account the variations of bioavailability induced by dietary shifts, possibly compromising the nutritional adequacy of the recommended diets and thus their sustainability. Hence, there is still a gap of knowledge regarding food choices that would both reduce the environmental impact of diets and ensure their nutritional adequacy.

Another issue related to reducing meat consumption, echoing the broader concept of sustainable food systems [21], is the consideration of the links between animal foods co-produced within the same food system. Meat production system actually generates several co-products. To illustrate, in France for instance, 35% of beef produced is of dairy type and is thus indirectly co-produced with milk [22]. Hence, a change in beef consumption, and thus in beef production, could affect milk production. To suggest realistic dietary changes to move towards sustainable food systems, links between foods belonging to the same production system should be considered. Previous studies on sustainable diets have not accounted for the co-production links between some foods, which may induce waste and raise several economic issues [23].

Using a diet optimization approach with linear and non-linear programming, the present study aimed to assess the impact of introducing nutrient bioavailability and co-production links considerations on the dietary changes needed ‒ especially regarding meat ‒ to improve diet sustainability. More specifically, the influence of accounting for diet-related bioavailability of four key nutrients (iron, zinc, protein, and provitamin A) and for animal co-production links was explored.

## Methods

### Population sample and dietary data

Dietary data from the second French individual and national study on food consumption (‘Etude Individuelle Nationale des Consommations Alimentaires’, or INCA2), a previously described [24] representative cross-sectional survey conducted between December 2005 and May 2007 by the former French Food Safety Agency (AFSSA), were used. The INCA2 study was conducted according to the guidelines laid down in the Declaration of Helsinki. Recruitment of participants was done by phone contact and oral consent was obtained, or not, during the call for practical reasons. All procedures involving human subjects/patients were approved by the ethic authority the French Data Protection Authority (Commission Nationale Informatique et Libertés). A total of 1342 foods and beverages were declared as consumed by the participants through 7-day food records. Foods were categorized—on the basis of French dietary guidelines, food habits and nutrient composition—into 8 food groups (e.g. ‘Fruit and vegetables’) and 27 food subgroups (e.g. ‘Fresh fruit’). Within the ‘Meat-fish-eggs’ group, the ‘Ruminant meat’ subgroup included pieces of meat from beef and lamb. After excluding children (age <18 y), energy under-reporters identified with Black equations [25], and individuals consuming hypocaloric meal substitutes, the final sample consisted of 773 men (age 49.0 ± 15.1) and 1126 women (age 45.8 ± 15.3).

### Food composition

The CIQUAL (French Information Center on Food Quality) food composition database associated with the INCA2 survey provided the nutrient content of all the foods declared as consumed. For mixed dishes, nutritional compositions were calculated based on ingredients proportions stated in the recipes from the INCA2 database. Further nutrients (provitamins and amino acids) as well as data on bioavailability modulators (phytates, tea, heme iron) were needed to take bioavailability into account. The food contents of α-carotene and β-cryptoxanthin (two other provitaminic A carotenoids besides β-carotene) were extracted from the U.S. Department of Agriculture National Nutrient Database for Standard Reference [26]. Phytate and amino acid contents were extracted from the WorldFood Dietary Assessment System 2 [27]. Heme iron (exclusively found in animal products) contents were extracted from the French Meat Information Center [28] and completed with Kongkachuichai *et al*.’s work for seafood and poultry [29]. Polyphenols from beverages were expressed as black tea equivalents with the conversion factors reported in Armah *et al*. [30]. All foods were screened and manually characterized as of plant or animal (meat, fish, eggs and dairy products) origin, and further distinguished by species when of animal origin.

### Bioavailability estimation

Bioavailability, as addressed here, refers to absorption rate in the case of iron and zinc, bioefficacy (accounting for absorption and conversion [31]) for provitamin A carotenoids, and quality (accounting for digestibility and biological value) for proteins. Bioavailability was estimated as previously described in Perignon et al. [32] using algorithms and food-dependent coefficients from the literature. Non-heme iron absorption (%) was estimated using the diet-based algorithm developed by Armah *et al*. [30] and was expressed as follows:

$$\begin{array}{l} Ln\left(non‑heme\;iron\;absorption\right)=6.294‑0.709\;ln\left(SF\right)\\ +\;0.119\;ln(C)+0.006ln(TMF+0.1)‑0.055ln\left(T+0.1\right)‑0.247ln\left(P\right)‑0.137ln\left(Ca\right)‑0.083ln\left(NH\right) \end{array}$$

where SF is serum ferritin (μg/L), C is vitamin C (mg), TMF is total meat plus fish (g), T is tea–coffee–wine (number of cups of black tea equivalents), P is phytate (mg), Ca is calcium (mg), and NH is nonheme iron (mg). Total meat quantity in the diets was calculated as the sum of meat (i.e. terrestrial livestock flesh) foods and meat products present as ingredients in mixed dishes.

Heme iron absorption (%) was estimated using Hallberg *et al*.’s equation [33]:

Log(hemeironabsorption)=1.9897−0.3092×logSF

where SF is serum ferritin (μg/L). In the absence of individual biologic values for serum ferritin (SF) in INCA2 participants, 30 μg/L was selected as SF reference. This value was used as a target by the European Food Safety Authority in their opinion statement on dietary reference values for iron [34]. The above equation led to an average heme iron absorption of 34.1%. Total iron absorption was calculated as the sum of non-heme and heme iron absorbed.

The amount of zinc available for absorption was calculated using Miller *et al*.’s algorithm as follows [35]:

$$TAZ=0.5\times \left\{0.13+TDZ+0.10(1+\frac{TDP}{1.2})-\sqrt{{\left(0.13+TDZ+0.10(1+\frac{TDP}{1.2})\right)}^{2}-4\times 0.13\times TDZ}\right\}$$

where TAZ is total absorbed zinc (mmol), TDZ is total dietary zinc (mmol) and TDP is total dietary phytates (mmol). Molar masses of 65.4 and 660 g.mol^-1^ were used for zinc and phytates, respectively.

Retinol equivalents from three provitamin A carotenoids (β-carotene, α-carotene and β-cryptoxanthin) were calculated using food- or food-group-specific bioefficacy data from the literature. On a weight basis, it is estimated that 21 μg of β-carotene from spinach is needed to finally obtain 1 μg of retinol [36], 14 μg from carrot [36,37], 12 μg from fruit [38,39], 27 μg from vegetables [40], 3.2 μg from maize [41], 3.8 μg from rice [42], and 9 μg from fats [43,44]. Given the lipophilic character of β-carotene, its coefficient in animal products was considered the same as in fats. Bioefficacies of α-carotene and β-cryptoxanthin were assumed to be half the bioefficacy of β-carotene (e.g. 42 μg from spinach needed to finally obtain 1 μg of retinol) [40]. In models where diet-related bioavailability was not taken into account, we only considered preformed retinol and β-carotene values from the CIQUAL database, and a coefficient of 6 was used for β-carotene regardless of food source [45,46].

Protein quality was calculated using the protein digestibility-corrected amino acid score (PDCAAS, in %) [47] at diet level as follows:

$$PDCAAS\;=\;PD\;\times {AAS}_{diet}$$

where PD is the protein digestibility (%) and AAS_diet_ is the amino acid score of the diet, i.e. the minimum amino acid score for indispensable amino acid i (AAS_i_) calculated as follows:

$${AAS}_{i}=\frac{indispensable\;amino\;acid\;i\;(mg)\;in\;1\;g\;of\;digested\;protein}{indispensable\;amino\;acid\;i\;(mg)\;in\;requirement\;pattern}$$

The adult pattern was used as reference pattern, defining the amino acid/protein ratio for each indispensable amino acid [47]. If greater than 1, AAS is truncated to 1 to calculate the PDCAAS. Protein digestibility was assigned by food source based on published values [47,48], i.e. meat and fish: 94%; milk and dairy: 95%; eggs: 97%; legumes: 85%; ready-to-eat cereals: 75%; whole bread: 92%; white bread: 97%; flour: 96%; whole grain cereals: 86%; refined wheat: 96%; refined rice: 89%; whole corn/maize: 86%; nuts and seeds: 91%; soy and derivatives: 86%. For other foods, protein digestibility was not taken into account (100%).

For mixed dishes, food-dependent bioavailability coefficients were calculated from their ingredients based on the recipes.

All the above-listed bioavailability-related algorithms and coefficients were introduced into the models where the bioavailability of the four key nutrients were taken into account.

### Environmental impact of foods

Environmental impact was estimated for a total of 402 foods including 391 foods previously identified as widely consumed among the French population [49], and 11 foods identified as having potential nutritional and/or environmental utility (e.g. soya-based products, some unrefined starchy foods, chestnuts). Three environmental impacts were estimated: GHGE (in carbon dioxide equivalents, CO_2_eq), atmospheric acidification (in sulfur dioxide equivalents, SO_2_eq) and marine eutrophication (in nitrogen equivalents, Neq). The values for these impacts were assigned by an environmental consulting firm (Bio by Deloitte, formerly Greenext Service, Paris, France) based on a hybrid input/output life cycle assessment method using international standards ISO 14040 [50] and 14044 [51] and French standards BP X30-323-0 [52] and BP X30-323-15 [53]. The methodology used to estimate the environmental impact values has been fully described by Bertoluci et al. [54]. Briefly, this cradle-to-grave approach combines French trade and production data as well as standard life cycle inventory to result in values reflecting average food products as consumed in the French market. The values include the whole life cycle of the foods, from farm production to usage and waste management of packaging, but exclude emissions arising from indirect land-use change and emissions from consumers’ transport from retail to home.

### Aggregation of dietary data

Dietary data were declared for 1342 foods but environmental data were only available for 402 of them. Intakes were thus aggregated into the 402 foods according to a previously-described method [12]. The mean observed consumption of those 402 foods has been calculated for men and women separately (OBS diets), based on individual food consumption data. Energy and nutrient intakes have been calculated by crossing the quantities of the 402 foods with their nutritional composition.

### Diet cost

Mean prices of each of the 402 foods were derived from a representative sample of 12,000 French households participating in the 2006 Kantar Worldpanel purchase-panel database [55] by dividing annual expenditure by the quantities purchased, as previously described [56].

### Diet optimization with linear and non-linear programming

A diet optimization approach with linear and non-linear programming was used to simultaneously consider all four dimensions of diet sustainability, i.e. nutrition, environment, cultural acceptability, and affordability. The mean observed diet in the French adult population was considered a proxy for a culturally acceptable diet, and therefore the lowest departure from the observed diet was pursued in the modeled diets. For each gender, three modeled diets, with environmental impacts (namely greenhouse gas emissions, eutrophication and acidification) each reduced by at least 30%, were obtained: i) in the NE (nutrition-environment) model, the fulfillment of RDA for all nutrients was imposed; ii) in the NEB (NE-bioavailability) model, nutritional adequacy was further ensured by accounting for diet-related bioavailability of iron, zinc, protein and provitamin A; iii) the NEB-CP (NEB-co-production) model additionally took into account the links between co-produced animal foods. The total cost of each modeled diet was constrained to remain below or equal the cost of the observed one, as a safeguard for affordability. The price and environmental impacts of individual foods were assumed to be constant.

#### Environmental constraints

A previous study of Perignon et al. [12] showed that French diet-related GHGE may be reduced by 30% while reaching nutritional adequacy without requiring major additional dietary shifts than those induced by meeting nutritional recommendations, and at a similar cost. Higher GHGE reductions (>30%) either impaired nutritional quality (in the absence of nutritional constraints) or required non-trivial dietary shifts, therefore compromising acceptability to reach nutritional adequacy. Considering these results, a reduction of dietary GHGE (reference year: 2007) of at least 30% was presently considered desirable and realistic. This value is in line with the European target of a 40% reduction of GHGE by 2030 (against 1990 as reference year) stated ahead of the COP21 conference [57]. Given that food-related GHGE are highly correlated with indicators of eutrophication and acidification [58,59], a reduction of at least 30% was also imposed to each of these two other indicators.

#### Nutritional constraints

Diet quality in the modeled diets was ensured by imposing a set of constraints on the dietary content of macronutrients, 5 fatty acids, 10 minerals, 11 vitamins, free sugars, cholesterol and fiber (see S1 **Table**), mainly based on the French RDA. Energy contents of modeled diets were set as equal to observed intakes (1937 kcal and 2602 kcal for women and men respectively), because the observed energy intake was close to French energy requirements for women and men (requirements at 2100 kcal and 2600 kcal for French women and men aged between 18–59 y [60]). Nutritional constraints differed between NE and both NEB and NEB-CP models for the four key nutrients. In the models accounting for diet-related bioavailability (NEB and NEB-CP diets), iron and zinc constraints were placed not on total intake but on quantities available for absorption (i.e. adjusted for bioavailability, estimated as described under ‘Bioavailability estimation’). Thus, for iron and zinc in NEB and NEB-CP models, the content available for absorption was constrained to meet the estimated level of physiological requirements for that nutrient, itself estimated by multiplying the RDA value by the mean bioavailability considered by the AFSSA to derive that RDA (i.e. 10% for iron and 25% for zinc [45]). For protein and vitamin A, the RDA was equally imposed in all modeled diets, but in NEB and NEB-CP diets the estimated contents of protein and total vitamin A (expressed as retinol) were corrected according to their food source.

Limiting nutritional and environmental constraints were identified as those constraints that were exactly met (i.e. exactly 100% of the value imposed by the constraint was reached in the modeled diet) and their strengths were assessed through their dual value [12,61].

#### Food quantities constraints

To avoid extreme deviations and thus unrealistic modeled diets, the food item, food subgroup and food group quantities were constrained to be lower than the 90^th^ percentile of the observed intakes. The 90^th^ percentile value used for the constraint on each food item was calculated by gender, from the observed food intakes of consumers only (i.e., excluding from the calculation non-consumers of the food item). The 90^th^ percentile values used for the constraint on each food group and subgroup were calculated by gender, from the observed food group and subgroup intakes of the whole population (both consumers and non-consumers of the food group or subgroup were included in the calculation)[62]. Total diet quantity (in g/d) was limited to within 80–120% of OBS quantity. Quantities of fortified foods, alcoholic beverages and mineral waters were constrained to less than or equal to OBS quantities.

#### Co-production constraints

Quantities of bovine meat and dairy products were co-constrained. Based on current French data, we imposed a maximal ratio of dairy products to bovine meat (from offspring and culled cows) as follows: bovine dairy protein (g) ≤ 0.43*bovine meat (g). The co-production constraints calculation is detailed in **S1 File**. Conversely, bovine meat quantities were not constrained by dairy product quantities given the possibility of eating meat from non-dairy breeds. The constraint relating milk to meat was applied through milk protein and bovine (but not ovine) meat in the diet, including the quantities incorporated in dishes as ingredients. Preliminary results on NEB models turned out to unrealistically favor blood sausage (a deli meat specialty made from pork blood) due to its high heme iron content. We therefore also imposed a maximal blood sausage-to-pork meat ratio, as follows: blood sausage (g) ≤ 0.13*pork meat (g) (see **S1 File**: Co-production constraints calculations for details).

#### Cost constraint

Total cost of the modeled diet was constrained to remain below or equal the cost of the observed diet, assuming that the current mean price of foods was constant.

#### Decision variables

Decision variables, i.e. variables whose value can be changed to optimize the objective function, were the quantities of the 402 food items representing the 1342 foods declared in the INCA2 survey (see Aggregation of dietary data section).

#### Objective function

For each gender, the objective function f expressed the departure from the mean observed diet at food-item level (j = 402 foods) and at food-group level (k = 8 groups). By minimizing this objective function, we encouraged both the food group (e.g. dairy products) and the food (e.g. mozzarella) quantities to stay as close as possible to the observed quantities, as follows:

$$f=\frac{1}{402}\times \sum_{j=1}^{402}\frac{ABS(Q_{j}-Q_{j,obs})}{Q_{j,obs}}+\frac{1}{8}\times \sum_{k=1}^{8}\frac{ABS(Q_{k}-Q_{k,obs})}{Q_{k,obs}}$$

where ABS is absolute value function, Q_j_ (respectively Q_k_) is quantity (in grams per day) of food j (resp. food group k) in the modeled diet, and Q_j, obs_ (resp. Q_k, obs_) is quantity (in grams per day) of food j (resp. food group k) in the mean observed diet.

The objective function f was transformed into a linear function via new decision variables as previously implemented by Darmon *et al*. [63]. Objective function and constraints of NE models were linear. For NEB and NEB-CP models, nonlinear programming was implemented due to the non-linearity of the constraints on absorbable zinc and iron, which means the algorithm may stop at a local optimum without finding the very best solution. To reach the optimal solution, we ran each model 1000 times and selected the one with the smallest objective function.

### Data analysis

For each gender, four diets were analyzed: the mean observed diet (OBS) and three modeled diets (NE, NEB and NEB-CP). Diet compositions (in g/d) were compared for each gender separately in terms of foods (n = 402), food subgroups (n = 27) and food groups (n = 8). ‘Total meat’ in the diets was calculated as the sum of meat (i.e. terrestrial livestock flesh) foods (e.g. “Cooked ham”) and meat products present as ingredients in mixed dishes (e.g. “Tinned cassoulet” contained 26.8% meat). ‘Total ruminant meat’ in the diets was calculated as the sum of all types of ruminant meat (beef, veal, mutton and lamb) and ruminant meat products present as ingredients in mixed dishes. SAS version 9.4 software was used for all models (*optmodel* procedure).

## Results

### Diet content

**Fig 1:**
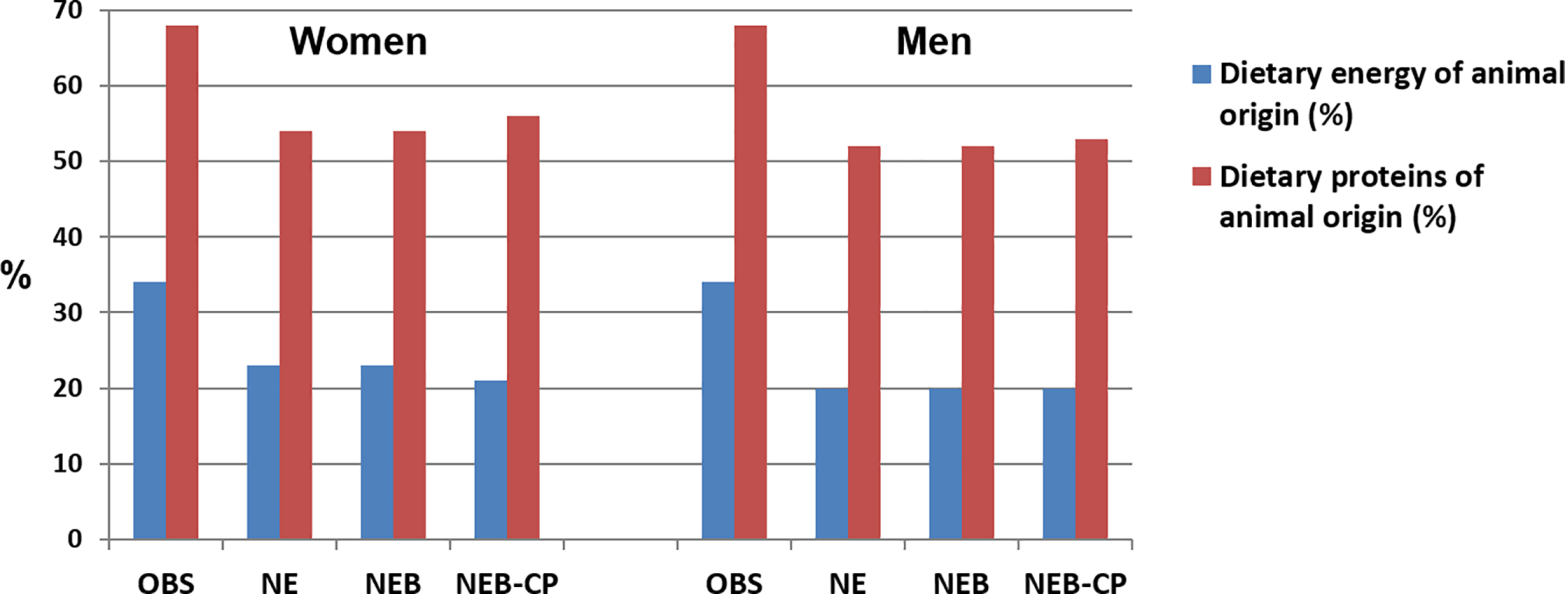
Share of dietary energy and proteins of animal origin in observed diet (OBS) and the three modeled diets (NE, NEB, NEB-CP) by gender. NE, nutrition-environment model; NEB, NE-bioavailability model; NEB-CP, NEB-co-production model; OBS, observed.

The share of energy and proteins of animal origin (**Fig 1**) was similar for the 3 modeled diets (approximately 1/5 of total energy, and 1/2 of protein) and lower than in OBS diet (approximately 1/3 of total energy, and 2/3 of protein). Changes observed at the food group and subgroup levels are described in the following paragraphs.

#### NE diets

**Fig 2:**
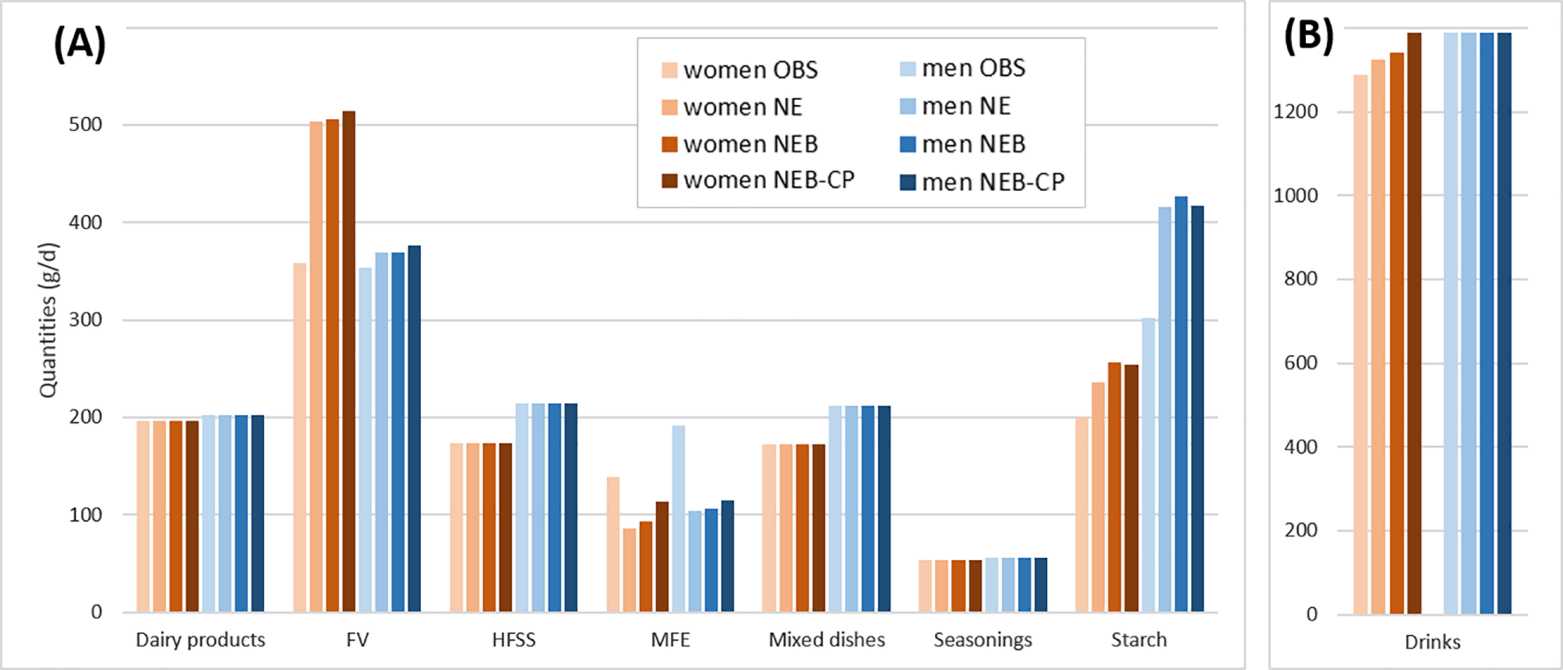
Food-group quantities in the observed diet (OBS) and in the three-modeled diets (NE, NEB, NEB-CP), for each gender. (A), Solid food groups; (B), Drinks. FV, fruit and vegetables; HFSS, high fat/sugar/salt foods; MFE, meat-fish-eggs NE, nutrition-environment model; NEB, NE-bioavailability model; NEB-CP, NEB-co-production model.

**Fig 3:**
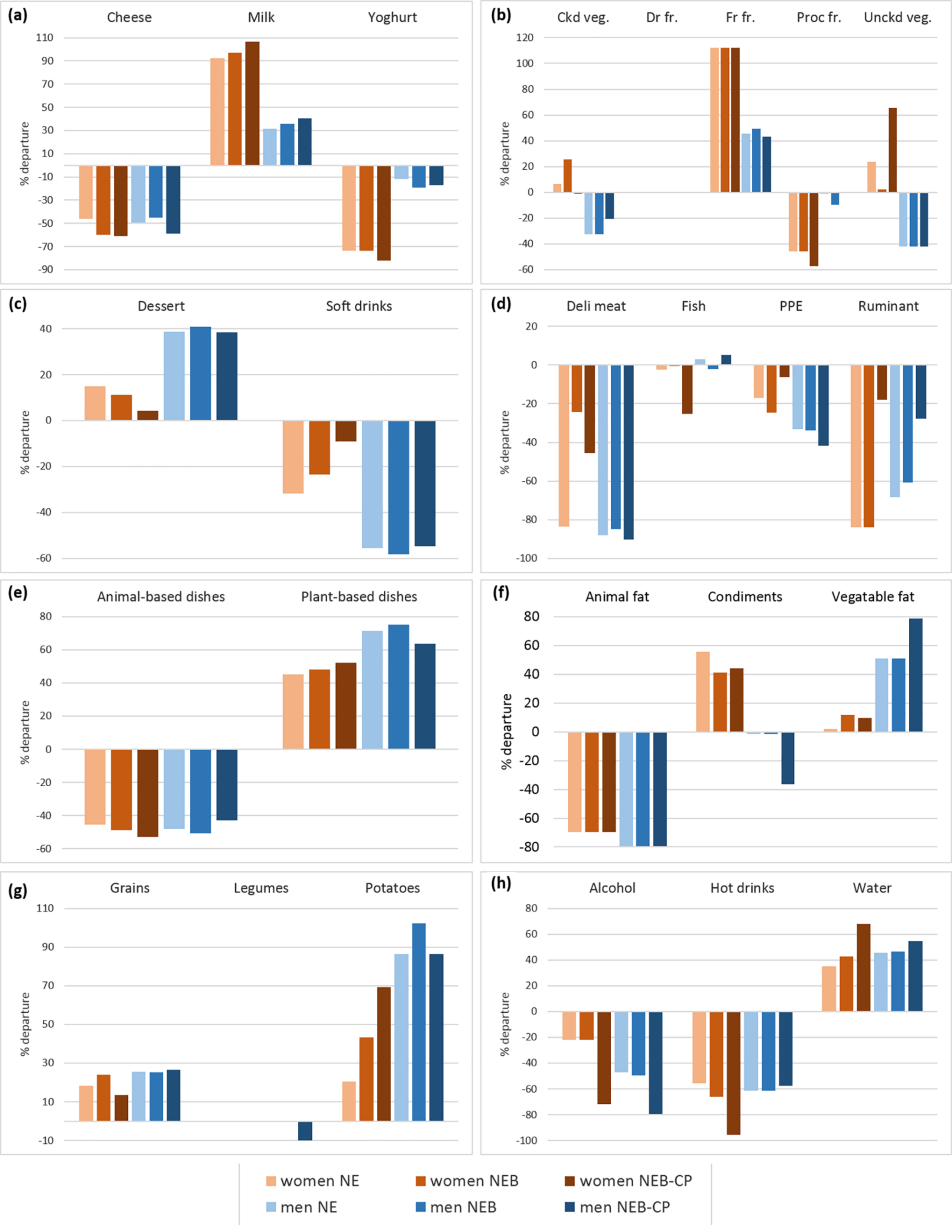
**Dietary changes induced by the three models (NE, NEB, NEB-CP), expressed in % variation of food subgroup quantities between modeled and observed diets, for each gender:** (a) dairy products; (b) fruit and vegetables; (c) high fat/sugar/salt foods; (d) meat-fish-eggs; (e) mixed dishes; (f) seasonings; (g) starch; (h) drinks. Food subgroups with quantities amounting to < 6 g in OBS and modeled diets (i.e. dried fruits, salty snacks and breakfast cereals) are not shown. Ckd veg, cooked vegetables; Dr fr, dried fruit; Fr fr, fresh fruit; NE, nutrition-environment model; NEB, NE-bioavailability model; NEB-CP, NEB-co-production model; PPE, pork-poultry-eggs; Proc fr, processed fruits; Unckd veg, uncooked vegetables.

In NE diets, the quantities of most food groups (‘Dairy products’, ‘High-fat/sugar/salt foods’, ‘Mixed dishes’, ‘Seasonings’ and ‘Drinks’) remained equal or close to quantities in OBS diets (**Fig 2 and S2 Table**). Quantities of the ‘Fruit and vegetables’ food group increased in NE compared to OBS diets, especially for women (+41% and +4% in women and men’s diets respectively). Quantities of the ‘Starch’ group also increased (+18 and +38% in women and men’s diets respectively) while quantities of ‘Meat-fish-eggs’ decreased by around 40% for both genders. At the more disaggregated food-subgroup level, the largest increases in NE compared to OBS diets were for ‘Milk’, ‘Fresh fruit’, ‘Vegetable-based dishes’ and ‘Potatoes’, and the largest decreases were for ‘Hot drinks’, ‘Deli meat’, ‘Ruminant meat’ and ‘Animal fats’ (**Fig 3 and S2 Table**). Compared to OBS diets, quantities of total meat (including meat contained in the animal-based mixed dishes) dropped by 78% and 68%, leading to a meat content of 25 g/d and 54 g/d, in the women’s and men’s NE diets, respectively (instead of 110 and 168 g/d in women’s and men’s OBS diets, respectively) (**Table 1**). More specifically, the quantities of total ruminant meat (including ruminant meat contained in the animal-based mixed dishes) dropped by 84% and 80%, leading to a content of 6g/d and 11g/d, in the women’s and men’s NE diets, respectively (instead of 36g/d and 54g/d in women’s and men’s OBS diets, respectively). The nutritional constraints that proved most difficult to fulfill were the upper constraints on saturated fatty acids and sodium contents and the lower constraints on fiber and carbohydrate contents.

**Table 1:** Bioavailability estimates and nutrient and bioavailability modulators contents in observed diet (OBS) and the three modeled diets (NE, NEB, NEB-CP) by gender^1^.

	Women				Men			
	OBS	NE	NEB	NEB-CP	OBS	NE	NEB	NEB-CP
**Bioavailability estimates**								
Iron absorption rate (%)	7.7	4.6	9.1	7.7	7.6	4.7	5.1	5.1
Zinc absorption rate (%)	30.7	25.7	27.0	25.8	25.9	24.0	23.9	23.8
Overall protein digestibility (%)	95.0	95.0	95.1	95.0	95.0	95.1	95.1	95.1
Amino Acid Score (untruncated to 1)	1.3	1.3	1.3	1.2	1.3	1.2	1.3	1.2
**Nutrient content expressed as percentage of recommended values**								
Total iron (% RDA)	68.3	**100.0**	110.5	129.1	162.2	**197.3**	195.5	199.5
Iron available for absorption (% absorbed iron requirement)	52.7	46.3	**100.0**	**100.0**	123.2	92.7	**100.0**	**101.0**
Total zinc (% RDA)	87.8	**100.0**	93.9	112.9	100.3	**100.0**	104.6	105.1
Zinc available for absorption (% absorbed zinc requirement)	107.9	102.8	**101.4**	**116.5**	104.0	96.1	**100.0**	**100.0**
Total protein (% RDA)	142.8	**115.7**	117.2	124.9	159.4	**121.0**	121.6	125.3
PDCAAS-corrected protein (% RDA)	135.6	110.0	**111.5**	**118.6**	151.4	115.1	**115.6**	**119.1**
Vitamin A, estimated with coefficients 6 and 12 for provitamin A carotenoids (% RDA)	178.5	**171.5**	175.6	220.8	159.3	**185.3**	207.8	154.9
Vitamin A, estimated with food-group-specific coefficients for provitamin A carotenoids (% RDA)	131.5	114.8	**113.7**	**135.1**	123.6	147.6	**169.7**	**117.2**
**Bioavailability modulators content**								
Heme iron (mg/d)	1.3	0.3	2.2	1.6	1.8	0.5	0.8	0.8
Phytates (mg/d)	795	1285	1162	1154	1071	1358	1331	1343
Vitamin C (mg/d)	97	110	110	139	95	110	110	110
Polyphenols from beverages (eq. cups of tea/d)	1.2	0.2	0.0	0.0	0.8	0.3	0.3	0.4
Calcium (mg/d)	854	900	900	900	1008	900	900	900
Total meat^2^ plus fish (g/d)	140	54	66	96	199	85	88	96
Total meat^2^ (g/d)	110	25	36	74	168	54	58	64
Total ruminant meat^3^ (g/d)	36	6	5	26	54	11	12	33

^1^Bold values indicate where a value ≥ 100% was imposed in the model. NE, nutrition-environment model; NEB, NE-bioavailability model; NEB-CP, NEB-co-production model; OBS, observed; PDCAAS, protein digestibility-corrected amino acid score; RDA, recommended dietary allowance.

^2^Total meat includes all types of meat (pork, poultry, ruminant meats) and meat products present as ingredients in mixed dishes.

^3^Total ruminant meat includes all types of ruminant meat (beef, veal, mutton and lamb) and ruminant meat products present as ingredients in mixed dishes.

#### NEB diets

Accounting for the diet-related bioavailability of the four key nutrients did not induce dramatic changes in terms of food composition. When comparing NEB to NE diets, the quantities did not depart by more than 9% for food groups (**Fig 2 and S2 Table**) and 25% for food subgroups (**Fig 3**). The only exception was for the ‘Deli meat’ subgroup, which increased by 358% in NEB compared to NE diet (from 3 to 14 g, driven by an increase of 11 g of blood sausage). Compared to OBS diets, quantities of total meat dropped by 67% and 66%, leading to a meat content of 36 g/d and 58 g/d, in the women’s and men’s NEB diets, respectively (**Table 1**). More specifically, the quantities of total ruminant meat dropped by 87% and 78%, leading to a content of 5g/d and 12g/d, in the women’s and men’s NEB diets, respectively.

#### NEB-CP models

In NEB-CP models, constraints were introduced to link bovine meat to milk protein and link blood sausage to pork meat. From NEB to NEB-CP diets, ‘Dairy products’ quantities remained stable for women and men whereas ‘Ruminant meat’ quantities increased by 405% (+21 g) and 83% (+15 g), and total meat increased by 106% (38 g) and 11% (6 g) for women and men, respectively. In order to fulfill all constraints, and especially maintain the 30% reduction of environmental impacts, changes had to occur for other food groups and for other food subgroups. From NEB to NEB-CP diets, alcoholic beverages were reduced by around 60% and hot drinks were reduced (only for women) by 88% (**Fig 3**). Compared to OBS diets, quantities of total meat dropped by 32% and 62%, leading to a meat content of 74 g/d and 64 g/d, in the women’s and men’s NEB-CP diets, respectively (**Table 1**). More specifically, the quantities of total ruminant meat dropped by 27% and 38%, leading to a content of 26g/d and 33g/d, in the women’s and men’s NEB-CP diets, respectively.

### Diet cost

OBS, NE, NEB and NEB-CP diets costs were 6.2€/d, 5.9€/d, 5.8€/d and 6.1€/d for women, and 8.1 €/d, 6.9€/d, 6.9€/d and 6.8€/d for men, respectively. Whatever the model applied, modeled diets cost less than OBS diets.

### Bioavailability estimations

**Table 1** reports the bioavailability estimates of the four key nutrients in the observed and modeled diets, and the contents in bioavailability modulators. Compared to the women’s OBS diet, modeled diets had higher total iron contents. However, compared with OBS diet, iron bioavailability was lower in the NE diet and equal or higher in the NEB and NEB-CP diets (**Table 1**). Heme iron contents were greater in in NEB and NEB-CP diets than OBS diet. Heme iron was sourced 26%, 18%, 88% and 54% by blood sausage and 34%, 19%, 3% and 20% by ‘Ruminant meat’ in the OBS, NE, NEB and NEB-CP diets respectively. For men and women, phytate contents were higher in the modeled diets than in OBS diet and higher in NE than in NEB and NEB-CP diets. Vitamin C was higher in women’s NEB-CP diet than NEB diet. Iron was especially limiting in women’s diets (high dual values). Protein and provitamin A were never limiting in both men and women (**Table 1**), where the share of energy and protein of animal origin was similar across NE, NEB and NEB-CP modeled diets and lower than in OBS diet (**Fig 1**).

### Contributions of food subgroups to environmental impacts

**Tables 2 and 3** report the per-food-subgroup contributions to environmental impacts of the observed and modeled diets in terms of GHGE and eutrophication. Acidification impacts were reduced by more than the 30% minimum reduction imposed to all modeled diets, showing that they were not levers of the dietary changes.

**Table 2:** Greenhouse gas emissions levels in g CO2eq (contribution to total GHGE in %) by food groups and food subgroups for the observed diet (OBS) and the three modeled diets (NE, NEB, NEB-CP), by gender^1^.

		Women				Men			
Group	Subgroup	OBS	NE	NEB	NEB-CP	OBS	NE	NEB	NEB-CP
**Fruit and vegetables**		443 (12.1)	443 (17.3)	440 (17.1)	426 (16.6)	423 (8.6)	409 (11.9)	405 (11.8)	379 (11.0)
	Cooked vegetables	134 (3.7)	136 (5.3)	160 (6.2)	117 (4.6)	133 (2.7)	85 (2.5)	85 (2.5)	99 (2.9)
	Dried fruit	4 (0.1)	4 (0.1)	4 (0.1)	4 (0.1)	4 (0.1)	4 (0.1)	4 (0.1)	4 (0.1)
	Fresh fruit	168 (4.6)	188 (7.3)	188 (7.3)	194 (7.6)	155 (3.2)	220 (6.4)	222 (6.5)	176 (5.1)
	Processed fruit	73 (2.0)	36 (1.4)	36 (1.4)	28 (1.1)	69 (1.4)	68 (2.0)	62 (1.8)	69 (2.0)
	Uncooked vegetables	64 (1.8)	79 (3.1)	52 (2.0)	82 (3.2)	62 (1.3)	31 (0.9)	31 (0.9)	31 (0.9)
**Starch**		289 (7.9)	309 (12.0)	325 (12.7)	311 (12.1)	434 (8.9)	515 (15.0)	529 (15.4)	514 (15.0)
	Grains	225 (6.1)	207 (8.1)	216 (8.4)	196 (7.6)	341 (7.0)	337 (9.8)	337 (9.8)	340 (9.9)
	Legumes	20 (0.6)	20 (0.8)	20 (0.8)	20 (0.8)	31 (0.6)	31 (0.9)	31 (0.9)	27 (0.8)
	Potatoes	44 (1.2)	82 (3.2)	88 (3.4)	96 (3.7)	62 (1.3)	146 (4.3)	161 (4.7)	147 (4.3)
**Dairy products**		408 (11.1)	306 (11.9)	290 (11.3)	271 (10.5)	459 (9.4)	375 (10.9)	377 (11.0)	354 (10.3)
	Cheese	136 (3.7)	74 (2.9)	53 (2.1)	53 (2.1)	210 (4.3)	110 (3.2)	120 (3.5)	88 (2.6)
	Milk	100 (2.7)	193 (7.5)	197 (7.7)	188 (7.3)	111 (2.3)	146 (4.2)	150 (4.4)	155 (4.5)
	Yoghurt	171 (4.7)	39 (1.5)	39 (1.5)	29 (1.1)	138 (2.8)	119 (3.5)	108 (3.1)	111 (3.2)
**High-fat/sugar/salt foods**		373 (10.2)	336 (13.1)	328 (12.8)	289 (11.3)	423 (8.6)	455 (13.3)	446 (13.0)	446 (13.0)
	Breakfast cereals	13 (0.4)	13 (0.5)	13 (0.5)	13 (0.5)	11 (0.2)	11 (0.3)	11 (0.3)	11 (0.3)
	Dessert	326 (8.9)	296 (11.5)	287 (11.2)	244 (9.5)	362 (7.4)	414 (12.1)	406 (11.8)	405 (11.8)
	Salty snacks	10 (0.3)	10 (0.4)	10 (0.4)	10 (0.4)	12 (0.2)	12 (0.3)	12 (0.3)	12 (0.3)
	Soft drinks	24 (0.7)	17 (0.6)	19 (0.7)	22 (0.8)	38 (0.8)	18 (0.5)	17 (0.5)	18 (0.5)
**Mixed dishes**		493 (13.4)	377 (14.7)	364 (14.2)	353 (13.7)	648 (13.2)	504 (14.7)	492 (14.4)	512 (14.9)
	Animal-based dishes	343 (9.4)	160 (6.2)	143 (5.6)	125 (4.9)	501 (10.2)	252 (7.4)	235 (6.9)	271 (7.9)
	Plant-based dishes	150 (4.1)	217 (8.4)	221 (8.6)	228 (8.9)	147 (3.0)	252 (7.3)	258 (7.5)	241 (7.0)
**Meat-fish-eggs**		1146 (31.2)	418 (16.3)	438 (17.1)	736 (28.7)	1646 (33.6)	650 (19.0)	682 (19.9)	883 (25.8)
	Deli meat	101 (2.8)	16 (0.6)	49 (1.9)	45 (1.7)	179 (3.7)	22 (0.6)	24 (0.7)	17 (0.5)
	Fish	155 (4.2)	130 (5.1)	132 (5.1)	74 (2.9)	154 (3.1)	161 (4.7)	137 (4.0)	149 (4.3)
	Pork-poultry-eggs	393 (10.7)	203 (7.9)	188 (7.3)	246 (9.6)	577 (11.8)	265 (7.7)	263 (7.7)	210 (6.1)
	Ruminant	497 (13.6)	69 (2.7)	69 (2.7)	370 (14.4)	736 (15.0)	202 (5.9)	257 (7.5)	506 (14.8)
**Seasonings**		154 (4.2)	78 (3.1)	84 (3.3)	82 (3.2)	171 (3.5)	100 (2.9)	100 (2.9)	116 (3.4)
	Animal fat	92 (2.5)	14 (0.5)	14 (0.5)	14 (0.5)	108 (2.2)	10 (0.3)	10 (0.3)	10 (0.3)
	Condiments	23 (0.6)	24 (0.9)	23 (0.9)	23 (0.9)	24 (0.5)	23 (0.7)	23 (0.7)	23 (0.7)
	Vegetable fat	39 (1.1)	41 (1.6)	47 (1.8)	45 (1.8)	39 (0.8)	66 (1.9)	66 (1.9)	83 (2.4)
**Drinks**		362 (9.9)	298 (11.6)	298 (11.6)	100 (3.9)	692 (14.1)	420 (12.2)	397 (11.6)	224 (6.5)
	Alcohol	108 (3.0)	84 (3.3)	84 (3.3)	26 (1.0)	436 (8.9)	227 (6.6)	217 (6.3)	83 (2.4)
	Hot drinks	140 (3.8)	174 (6.8)	174 (6.8)	35 (1.4)	150 (3.1)	87 (2.5)	74 (2.2)	88 (2.6)
	Water	114 (3.1)	40 (1.6)	40 (1.6)	40 (1.6)	105 (2.2)	106 (3.1)	106 (3.1)	53 (1.6)
**Total**	**Sum**	**3667 (100)**	**2567 (100)**	**2567 (100)**	**2567 (100)**	**4896 (100)**	**3428 (100)**	**3428 (100)**	**3428 (100)**

^1^NE, nutrition-environment model; NEB, NE-bioavailability model; NEB-CP, NEB-co-production model; OBS, observed.

**Table 3:** Eutrophication levels in g Neq (contribution to total eutrophication in %) by food groups and food subgroups for the observed diet (OBS) and the three-modeled diets (NE, NEB, NEB-CP), by gender^1^.

		Women				Men			
Group	Subgroup	OBS	NE	NEB	NEB-CP	OBS	NE	NEB	NEB-CP
**Fruit and vegetables**		1.87 (11.7)	1.87 (16.7)	1.88 (16.8)	1.64 (14.7)	1.89 (8.7)	1.74 (11.5)	1.72 (11.3)	1.73 (11.3)
	Cooked vegetables	0.92 (5.8)	0.79 (7.1)	0.87 (7.8)	0.49 (4.3)	0.95 (4.4)	0.61 (4.0)	0.61 (4.0)	0.62 (4.1)
	Dried fruit	0.02 (0.2)	0.02 (0.2)	0.02 (0.2)	0.02 (0.2)	0.03 (0.2)	0.03 (0.2)	0.03 (0.2)	0.03 (0.2)
	Fresh fruit	0.45 (2.8)	0.68 (6.1)	0.68 (6.1)	0.77 (6.9)	0.43 (2.0)	0.69 (4.5)	0.69 (4.6)	0.65 (4.3)
	Processed fruit	0.30 (1.9)	0.13 (1.1)	0.13 (1.1)	0.09 (0.8)	0.30 (1.4)	0.29 (1.9)	0.26 (1.7)	0.30 (2.0)
	Uncooked vegetables	0.17 (1.1)	0.25 (2.2)	0.18 (1.6)	0.27 (2.4)	0.17 (0.8)	0.12 (0.8)	0.12 (0.8)	0.12 (0.8)
**Starch**		1.66 (10.4)	2.14 (19.2)	2.23 (20.0)	2.18 (19.5)	2.52 (11.6)	3.48 (22.9)	3.55 (23.3)	3.47 (22.8)
	Grains	1.25 (7.8)	1.63 (14.6)	1.68 (15.0)	1.57 (14)	1.93 (8.9)	2.57 (16.9)	2.58 (17)	2.60 (17.0)
	Legumes	0.20 (1.2)	0.20 (1.8)	0.20 (1.8)	0.20 (1.8)	0.30 (1.4)	0.3 (2.0)	0.30 (2.0)	0.27 (1.7)
	Potatoes	0.22 (1.4)	0.31 (2.8)	0.36 (3.2)	0.41 (3.7)	0.29 (1.3)	0.61 (4.0)	0.67 (4.4)	0.61 (4.0)
**Dairy products**		0.71 (4.5)	0.55 (4.9)	0.52 (4.6)	0.48 (4.3)	0.82 (3.8)	0.67 (4.4)	0.67 (4.4)	0.63 (4.1)
	Cheese	0.24 (1.5)	0.13 (1.1)	0.09 (0.8)	0.09 (0.8)	0.38 (1.8)	0.19 (1.3)	0.21 (1.4)	0.15 (1.0)
	Milk	0.18 (1.2)	0.36 (3.2)	0.36 (3.3)	0.35 (3.1)	0.20 (0.9)	0.27 (1.8)	0.28 (1.8)	0.29 (1.9)
	Yoghurt	0.29 (1.8)	0.06 (0.5)	0.06 (0.5)	0.04 (0.4)	0.23 (1.1)	0.20 (1.3)	0.19 (1.2)	0.19 (1.3)
**High-fat/sugar/salt foods**		1.38 (8.6)	1.44 (12.9)	1.42 (12.7)	1.13 (10.1)	1.54 (7.1)	1.86 (12.2)	1.83 (12.0)	1.89 (12.4)
	Breakfast cereals	0.09 (0.6)	0.09 (0.8)	0.09 (0.8)	0.09 (0.8)	0.07 (0.3)	0.07 (0.5)	0.07 (0.5)	0.07 (0.5)
	Dessert	1.18 (7.4)	1.26 (11.3)	1.23 (11.0)	0.94 (8.4)	1.30 (6.0)	1.68 (11.1)	1.65 (10.8)	1.72 (11.3)
	Salty snacks	0.04 (0.2)	0.04 (0.3)	0.04 (0.3)	0.04 (0.3)	0.05 (0.2)	0.05 (0.3)	0.05 (0.3)	0.05 (0.3)
	Soft drinks	0.07 (0.4)	0.05 (0.4)	0.05 (0.5)	0.06 (0.6)	0.11 (0.5)	0.05 (0.4)	0.05 (0.3)	0.05 (0.4)
**Mixed dishes**		1.82 (11.4)	1.12 (10.0)	1.06 (9.5)	1.02 (9.2)	2.57 (11.8)	1.67 (11.0)	1.63 (10.7)	1.71 (11.2)
	Animal-based dishes	1.49 (9.4)	0.67 (6.0)	0.61 (5.4)	0.55 (5.0)	2.24 (10.3)	1.15 (7.6)	1.10 (7.2)	1.21 (8.0)
	Plant-based dishes	0.33 (2.1)	0.45 (4.0)	0.46 (4.1)	0.47 (4.2)	0.33 (1.5)	0.52 (3.4)	0.53 (3.5)	0.50 (3.3)
**Meat-fish-eggs**		6.53 (40.9)	2.70 (24.2)	2.76 (24.7)	3.87 (34.7)	9.65 (44.4)	3.84 (25.2)	3.94 (25.9)	4.27 (28)
	Deli meat	0.84 (5.3)	0.14 (1.2)	0.32 (2.8)	0.32 (2.9)	1.48 (6.8)	0.18 (1.2)	0.19 (1.3)	0.14 (0.9)
	Fish	0.46 (2.9)	0.44 (3.9)	0.44 (3.9)	0.17 (1.5)	0.44 (2.0)	0.54 (3.6)	0.50 (3.3)	0.63 (4.2)
	Pork-poultry-eggs	3.82 (24.0)	1.94 (17.4)	1.81 (16.2)	2.34 (21.0)	5.64 (25.9)	2.56 (16.8)	2.55 (16.7)	2.04 (13.4)
	Ruminant	1.41 (8.8)	0.19 (1.7)	0.19 (1.7)	1.04 (9.3)	2.09 (9.6)	0.55 (3.6)	0.69 (4.6)	1.45 (9.5)
**Seasonings**		0.75 (4.7)	0.60 (5.4)	0.60 (5.4)	0.60 (5.4)	0.77 (3.5)	0.90 (5.9)	0.88 (5.8)	0.86 (5.7)
	Animal fat	0.17 (1.1)	0.03 (0.2)	0.03 (0.2)	0.03 (0.2)	0.20 (0.9)	0.02 (0.1)	0.02 (0.1)	0.02 (0.1)
	Condiments	0.06 (0.4)	0.07 (0.6)	0.06 (0.6)	0.06 (0.5)	0.06 (0.3)	0.06 (0.4)	0.06 (0.4)	0.06 (0.4)
	Vegetable fat	0.52 (3.3)	0.50 (4.5)	0.51 (4.5)	0.51 (4.6)	0.51 (2.3)	0.81 (5.3)	0.80 (5.2)	0.78 (5.1)
**Drinks**		1.23 (7.7)	0.76 (6.8)	0.71 (6.3)	0.25 (2.2)	2.00 (9.2)	1.07 (7.0)	1.01 (6.6)	0.67 (4.4)
	Alcohol	0.28 (1.7)	0.22 (2.0)	0.22 (2.0)	0.07 (0.7)	1.12 (5.2)	0.56 (3.7)	0.54 (3.6)	0.23 (1.5)
	Hot drinks	0.77 (4.8)	0.47 (4.2)	0.42 (3.8)	0.11 (1.0)	0.70 (3.2)	0.33 (2.2)	0.29 (1.9)	0.35 (2.3)
	Water	0.19 (1.2)	0.07 (0.6)	0.07 (0.6)	0.07 (0.6)	0.17 (0.8)	0.17 (1.1)	0.17 (1.1)	0.09 (0.6)
**Total**	**Sum**	**15.96 (100)**	**11.17 (100)**	**11.17 (100)**	**11.17 (100)**	**21.75 (100)**	**15.23 (100)**	**15.23 (100)**	**15.23 (100)**

^1^NE, nutrition-environment model; NEB, NE-bioavailability model; NEB-CP, NEB-co-production model; OBS, observed.

The ‘Ruminant meat’ subgroup was the lead contributor to GHGE in the observed diets; its contribution dropped in NE and NEB diets but climbed back up in NEB-CP diets. In NEB-CP diets, the contributions of ‘Drinks’ to total GHGE dropped compared to NEB diets, especially due to decreased quantities of alcoholic beverages and hot drinks (**Table 2**). ‘Pork-poultry-eggs’ was lead contributor to eutrophication impact of OBS diets (24–26%) and still contributed to 16–17% of eutrophication impacts of the NE and NEB modeled diets (**Table 3**).

## Discussion

To our knowledge, this is the first study to quantitatively consider diet-related nutrient bioavailability and co-production relationships between animal foods when designing more sustainable diets. Reducing the environmental impact of diets by at least 30% while respecting the RDAs (NE model) required an increase in quantities of fruit & vegetables and starches, and a severe cut in total quantities of meat (reduction by around 70%), especially ruminant meat (reduction by around 70–80%). Further integration of the bioavailability of key nutrients (NEB model) did not change this overriding need to reduce total quantities of meat and especially ruminant meat, but food substitutions occurred, including within the meat category, in order to meet absorbable iron requirements for women (inclusion of more blood sausage). When co-production links were integrated (NEB-CP model), reductions in meat were less severe but total quantities of meat still decreased by around 30–60% *vs*. the observed diet, and by 30–40% for ruminant meat.

Previous studies assessed the environmental impact of observed or recommended diets [64–67]. However, the scenarios assessed in these studies were based on a-priori decisions about dietary changes, possibly neglecting cultural acceptability, which is an important dimension of diet sustainability. Diet optimization with linear and non-linear programming is a valuable tool for identifying nutritious yet environmentally-friendlier diets, starting from real food choices. Based on current mean women’s UK diet, MacDiarmid et al. used linear programming to model the “Livewell 2020 Plate”, which had a 25% reduction in GHGE (compared to 1990 baseline) and was able to fulfill a set of nutritional constraints [68]. Compared to the mean diet, the Livewell diet contained more fruit and vegetables, slightly more starch and less meat (the weight contribution of “meat and meat dishes” decreased from 16% in the mean observed diet to 4% “meat only” in the modeled diet, with a preference for chicken) and also less high fat and/or sugar foods. For a roughly 30% reduction of GHGE for women, red meat quantities decreased by more than 50% and dairy and eggs decreased by around 50% while fruit and vegetables practically doubled and cereals increased by 50%. In the Netherlands, van Dooren et al. also used linear programming to impose a 50% reduction of GHGE and a complete set of nutritional constraints which led to an almost vegetarian food basket that was cheaper than the mean observed diet [14]. In a previous modeling study based on the same French dietary data as here, we showed that it is possible to model diets with a 30% reduction of dietary GHGE while meeting nutritional recommendations without impairing diet affordability [12]. The modeled diet contained more fruit and vegetables and starch and less meat than the mean observed diet (around 75% less ruminant meat and deli meat). These studies thus converge toward the necessity to reduce meat, especially ruminant meat, in order to improve diet sustainability. However, such conclusion might be challenged by a number of limitations identified in the above modeling studies, such as: allowing the introduction of fortified foods [12], taking into account only one environmental indicator [12,13,68], including a limited number of food variables [13,68] or a limited number of nutritional constraints [13], and not taking into account the differential bioavailability of key nutrients nor co-production links between foods [12–14,68]. We therefore conducted this study to try to overcome these limits in order to assess whether the conclusion on the necessity to reduce meat to improve diet sustainability would still remain valid.

The dietary changes induced by the introduction of bioavailability in the models were mostly driven by the necessity to meet iron requirements in women’s diet. Given that iron content of the OBS diet fell far short of women’s iron requirements, an increase of blood sausage quantity in the NEB diet occurred that was clearly related to the need for heme iron, which is more readily absorbed than non-heme iron. In the women NEB diet, not only was the quantity of heme-iron increased, but also was the absorption of non-heme iron promoted by improving the balance between enhancers and inhibitors of non-heme iron absorption (*e*.*g* reducing phytates as compared to NE diet). In the NEB-CP diet, quantity of blood sausage was restrained by its link with quantity of pork meat: in order to still fulfill requirements despite this new constraint, dietary changes occurred that simultaneously increased total iron and vitamin C (an iron absorption enhancer). This study suggests that for zinc, protein and vitamin A, in the context of French diet, the diversity of sources is such that even when switching to smaller quantities of animal products, animal and plant sources can be complementarily combined to provide quantitatively and qualitatively sufficient amounts of these nutrients. Incorporating the links between co-produced animal foods in the NEB-CP model limited the drastic decrease in ruminant meat and increase in blood sausage that occurred in the NEB diet. Maintaining relatively high quantities of ruminant meat in the NEB-CP diet made it necessary to compensate for the corresponding environmental burden by introducing other dietary changes, such as decreasing the amounts of alcoholic beverages and hot drinks. This ties in with Hendrie et al. [69] and Vieux et al. [49] who reported the environmental burden of non-core foods in the diet. The impact of co-production links shown here suggests that such relationships should be integrated and expanded in future models. This study also highlights the ‘Pork-poultry-eggs’ food subgroup as a big driver of eutrophication impact. Hence, favoring meat from monogastric animals by replacing beef by pork or poultry meat, as proposed in some studies [70,71], may not address other serious environmental issues despite having beneficial impact on climate change.

Despite bringing novel findings, this study also carries limitations. It would still be of interest to consider other environmental impacts in future models, such as water footprint, land-use or biodiversity, as Meier et al. showed how one environmental indicator can evolve in the opposite direction to others (namely water footprint and GHGE in vegetarian/vegan diets) [72]. However, Gephart et al. suggested that minimizing each of the different footprint indicators (carbon, nitrogen, water and land footprints) yields similar diets [73]. Note too that beef does present some advantages that were not taken into account here, especially as it values inedible resources (and marginal lands) such as grass [74] and helps maintain pastureland and thus participates in biodiversity conservation [75] and carbon storage [76–78]. Diet optimization with linear and non-linear programming also carries limitations. In particular, the food changes modeled are primarily oriented by the objective function, which is arbitrarily defined, based on preconceived views of the modeler. In the present study, in order to remain close to current mean food consumption levels in the population, departure from the observed diet in terms of food content was minimized, but equal weighting was given to each food, which might not be considered as acceptable by the consumer. Other decision could have been made. For instance, Green et al. imposed a more limited number of nutritional requirements as constraints in their models but accounted for budget shares and price elasticities in their objective function to minimize the loss of consumer welfare [13]. Nevertheless, it remains that, whatever the form of the objective function, the departure might be too large to be acceptable, even if it is minimized. We used mean French diets as our point of departure, what hindered the inter-individual variability in terms of food consumption patterns and nutritional requirements. Developing individual optimized diets based on individual food consumption and nutritional status would help to identify tailored and more robust strategies to achieve sustainability targets [79]. Moreover, GHGE estimates were mean values representative of the current French situation in terms of production, processes and distribution modes, but that did not allow assessing the variability of the environmental impacts. Future research would benefit from development of datasets for environmental indicators that include different estimates by food item, depending on the source (eg, locally produced vs air shipped) and mode of production (eg, open-field vs greenhouse, organic vs conventional) of the food. In addition, diet cost was based on current mean food prices, without taking into account that changes in food patterns will change demand, hence food prices. Similarly, we did consider that the environmental impacts of each food were constant although changes in food demand can also impact food production chains, hence modifying the environmental impacts of foods. Such consequential effects were beyond the scope of the present study but future models could be improved, for instance by introducing price elasticities in order to better account for loss of consumer welfare. Consequential life cycle analysis would also have improved our models, as there are different carbon intensities between meat from dairy systems and meat from meat-only systems [80]. Moreover, co-production links could be considered more finely in the models by taking into account the quantities of dairy and meat available on French market given the current import and export levels.

Bioavailability appraisal could also be improved. For instance, casein was not explicitly included in the algorithm in the present study, although it can inhibit iron absorption [81]. However, this algorithm was chosen because it has been developed based on complete diet datasets, and confronted to a measure of iron absorption using an extrinsic radiolabeling technique [30]. Iron bioavailability was considered in our models via estimations assuming the single-same serum ferritin level of 30 μg/L (a value reflecting adequate iron stores) [34], and we did not therefore account for the variability related to host factors, whereas serum ferritin level is known to be the biggest explanatory factor of iron absorption [82]. In addition, calcium bioavailability was not considered here, because, to our knowledge, no algorithm has yet been developed to predict calcium absorption from food consumption data. Finally, this study is focusing on the French diet. We can expect similar shifts for countries with similar dietary patterns, but caution should be exercised when trying to extrapolate the present results to other populations, especially in developing countries, where micronutrient deficiencies are still prevalent.

The present modeling study propose diets that can theoretically achieve nutritional adequacy at reduced environmental impact, while being culturally acceptable for the French population, and accounting for diet-related bioavailability of four key nutrients and for co-production links. Whatever the model, diets with less meat were strictly required to improve sustainability.

## Supporting information

S1 Table Values of energy and nutritional constraints applied in the models. (PDF)

S2 Table Food groups and food subgroups quantities in g/d (in kcal/d) for the observed diet (OBS) and the three-modeled diets (NE, NEB, NEB-CP), by gender. (PDF)

S1 File Co-production constraints calculations. (PDF)
