# Supplementary material for: Integrating nutrient bioavailability and co-production links when identifying sustainable diets: How low should we reduce meat consumption?
Source: PLoS One. 2018 Feb 14;13(2):e0191767. doi: 10.1371/journal.pone.0191767 (PMC5812584; doi:10.1371/journal.pone.0191767)
Supplement: S1 Table — (PDF) [file pone.0191767.s001.pdf]

S1 Table. Values of energy and nutritional constraints applied in the models

Energy and nutrients	Constraint	Reference
Energy (kcal/d)	= observed	
Proteins (% of energy)	10–20	(1)
Carbohydrates (% of energy)	50–75	(2)
Total fat (% of energy)	20–35	(3)
α-linolenic acid (% of energy)	≥ 0.5	(3)
Linoleic acid (% of energy)	2.5–9	(3)
EPA + DHA (g/d)	≥ 0.25	(3)
Polyunsaturated fatty acids (% of energy)	6–11	(3)
Saturated fatty acids (% of energy)	≤ 10	(4)
Free sugars (% of energy)	≤ 10	(4)
Sodium (mg/d)	1,500–2,759 (men)/2,365 (women)	(5)
Cholesterol (mg/d)	≤ 300	(4)
Fiber (g/d)	≥ 30	(6)
Vitamin A (µg retinol equivalent)	800-1800 (men), 600-1600 (women)	(6)
Thiamine (mg/d)	≥ 1.3 (men), 1.1 (women)	(6)
Riboflavin (mg/d)	≥ 1.6 (men), 1.5 (women)	(6)
Niacin (mg/d)	14–47 (men), 11–47 (women)	(6)
Vitamin B12 (µg/d)	≥ 2.4	(6)
Pantothenic acid (mg/d)	≥ 5	(6)
Vitamin B6 (mg/d)	1.8–6.8 (men), 1.5–6.8 (women)	(6)
Folates (µg/d)	330–1500 (men), 300–1500 (women)	(6)
Ascorbic acid (mg/d)	110–1110	(6)
Vitamin D (µg/d)	≥ observed	
Vitamin E (mg/d)	12–52	(6)
Zinc (mg/d)	12–50 (men), 10–50 (women)	(6)
Absorbed zinc (mg/d)	3 (men), 2.5 (women)	
Selenium (µg/d)	60–350 (men), 50–350 (women)	(6)
Potassium (mg/d)	≥ 3100	(6)
Phosphorus (mg/d)	≥ 750	(6)

Magnesium (mg/d)	420–770 (men), 360–710 (women)	(6)
Iodine (μg/d)	≥ 150	(6)
Iron (mg/d)	9–28 (men), 16–28 (women)	(6)
Absorbed iron (mg/d)	≥ 0.9 (men), 1.6 (women)	
Copper (mg/d)	≥ 2 (men), 1.5 (women)	(6)
Copper (mg/kgBW)	≤ 0.5	
Calcium (mg/d)	900–2000	(6)

References

1. WHO/FAO/UNU. Protein and amino acid requirements in human nutrition: Repot of a joint FAO/WHO/UNU expert consultation. Geneva; 2002.
2. Mann J, Cummings JH, Englyst HN, Key T, Liu S, Riccardi G, Summerbell C, Uauy R, van Dam RM, Venn B, et al. FAO/WHO scientific update on carbohydrates in human nutrition: conclusions. Eur J Clin Nutr. 2007;61 Suppl 1:S132-7.
3. FAO/WHO. Interim summary of conclusions and dietary recommendations on total fat & fatty acids. 2008.
4. Joint WHO/FAO Expert Consultation. Diet, nutrition and the prevention of chronic diseases. World Health Organ Tech Rep Ser. 2003;916:i–viii, 1-149, backcover.
5. Pietinen P, Valsta LM, Hirvonen T, Sinkko H. Labelling the salt content in foods: a useful tool in reducing sodium intake in Finland. Public Health Nutr. Proquest; 2008;11:335–40.
6. Martin. Apports nutritionnels conseillés pour la population française, 3ième édition (Recommended dietary references for French population, 3rd edition). 3rd ed. Tec et Doc Lavoisier; 2000. 605 p.
