# Supplementary material for: Integrating nutrient bioavailability and co-production links when identifying sustainable diets: How low should we reduce meat consumption?
Source: PLoS One. 2018 Feb 14;13(2):e0191767. doi: 10.1371/journal.pone.0191767 (PMC5812584; doi:10.1371/journal.pone.0191767)
Supplement: S2 Table — (PDF) [file pone.0191767.s002.pdf]

S2 Table. Food groups and food subgroups quantities in g/d (in kcal/d) for the observed diet (OBS) and the three-modeled diets (NE, NEB, NEB-CP), by gender[1]

Group	Subgroup	Women				Men			
		OBS	NE	NEB	NEB-CP	OBS	NE	NEB	NEB-CP
**Dairy products**		196 (194)	196 (140)	196 (130)	196 (115)	202 (236)	202 (179)	202 (185)	202 (167)
	Cheese	27 (89)	14 (48)	11 (36)	10 (36)	41 (136)	21 (72)	22 (78)	17 (58)
	Milk	82 (38)	159 (73)	162 (75)	170 (66)	90 (43)	118 (56)	122 (58)	126 (60)
	Yoghurt	87 (67)	23 (19)	23 (19)	15 (13)	71 (56)	62 (51)	57 (49)	59 (49)
**Fruit and vegetables**		358 (169)	503 (273)	506 (256)	514 (272)	354 (172)	369 (221)	369 (221)	376 (223)
	Cooked vegetables	78 (29)	83 (28)	98 (32)	77 (24)	76 (29)	51 (20)	51 (20)	60 (22)
	Dried fruit	2 (7)	2 (7)	2 (7)	2 (7)	2 (11)	2 (11)	2 (11)	2 (11)
	Fresh fruit	143 (76)	304 (164)	304 (164)	304 (167)	146 (81)	212 (143)	217 (146)	209 (143)
	Processed fruit	75 (40)	41 (26)	41 (26)	32 (22)	69 (35)	69 (34)	63 (32)	69 (35)
	Uncooked vegetables	60 (16)	74 (48)	61 (26)	99 (51)	61 (17)	35 (13)	35 (13)	35 (13)
**High-fat/sugar/salt foods**		173 (407)	173 (422)	173 (404)	173 (411)	214 (455)	214 (565)	214 (571)	214 (578)
	Breakfast cereals	5 (21)	5 (21)	5 (21)	5 (21)	4 (18)	4 (18)	4 (18)	4 (18)
	Dessert	112 (348)	129 (371)	125 (350)	117 (355)	121 (382)	168 (511)	171 (518)	168 (524)
	Salty snacks	3 (13)	3 (13)	3 (13)	3 (13)	3 (16)	3 (16)	3 (16)	3 (16)
	Soft drinks	53 (25)	36 (18)	41 (20)	48 (23)	85 (39)	38 (19)	35 (18)	38 (20)
**Meat-fish-eggs**		139 (253)	86 (133)	93 (153)	114 (187)	191 (373)	104 (175)	107 (182)	115 (184)
	Deli meat	19 (57)	3 (10)	14 (38)	10 (31)	32 (100)	4 (15)	5 (17)	3 (12)
	Fish	28 (38)	28 (38)	28 (39)	21 (29)	28 (40)	29 (43)	28 (41)	30 (45)
	Pork-poultry-eggs	60 (100)	50 (78)	45 (68)	57 (87)	84 (147)	56 (93)	56 (93)	49 (71)
	Ruminant	31 (58)	5 (7)	5 (7)	26 (41)	46 (86)	15 (24)	18 (30)	33 (55)
**Mixed dishes**		172 (194)	172 (133)	172 (128)	172 (121)	212 (270)	212 (191)	212 (187)	212 (192)
	Animal-based dishes	86 (156)	47 (88)	44 (82)	40 (74)	127 (231)	66 (139)	63 (134)	73 (141)

	Plant-based dishes	87 (38)	126 (45)	129 (46)	132 (47)	85 (39)	146 (52)	150 (53)	140 (50)
**Seasonings**		53 (257)	53 (194)	53 (214)	53 (208)	56 (273)	56 (293)	56 (292)	56 (350)
	Animal fat	13 (77)	4 (8)	4 (8)	4 (8)	15 (92)	3 (6)	3 (6)	3 (6)
	Condiments	16 (9)	25 (10)	23 (10)	23 (9)	18 (10)	18 (10)	18 (10)	12 (10)
	Vegetable fat	23 (170)	24 (176)	26 (196)	26 (191)	23 (170)	35 (277)	35 (276)	41 (334)
**Starch**		200 (395)	236 (523)	256 (519)	254 (554)	302 (618)	416 (798)	426 (823)	417 (804)
	Grains	140 (328)	166 (416)	174 (404)	159 (429)	221 (525)	278 (603)	277 (603)	280 (609)
	Legumes	9 (10)	9 (10)	9 (10)	9 (10)	14 (15)	14 (15)	14 (15)	12 (14)
	Potatoes	51 (57)	61 (96)	73 (105)	86 (115)	67 (78)	125 (181)	136 (205)	125 (181)
**Drinks**		1289 (68)	1325 (119)	1342 (134)	1387 (69)	1389 (206)	1389 (181)	1389 (142)	1389 (105)
	Alcohol	63 (50)	50 (39)	49 (39)	18 (15)	256 (182)	135 (115)	129 (111)	53 (50)
	Hot drinks	421 (17)	186 (80)	142 (95)	18 (54)	369 (24)	143 (65)	141 (31)	156 (55)
	Water	805 (0)	1089 (0)	1150 (0)	1352 (0)	764 (0)	1111 (0)	1118 (0)	1180 (0)
**Total**		2581 (1938)	2745 (1938)	2791 (1938)	2864 (1938)	2920 (2603)	2963 (2603)	2975 (2603)	2982 (2603)

[1]NE, nutrition-environment model; NEB, NE-bioavailability model; NEB-CP, NEB-co-production model; OBS, observed
