# Supplementary material for: Integrating nutrient bioavailability and co-production links when identifying sustainable diets: How low should we reduce meat consumption?
Source: PLoS One. 2018 Feb 14;13(2):e0191767. doi: 10.1371/journal.pone.0191767 (PMC5812584; doi:10.1371/journal.pone.0191767)
Supplement: S1 File — (PDF) [file pone.0191767.s003.pdf]

S1 File: Co-production constraints calculations

Dairy products and bovine meat

Producing milk necessarily coproduces meat: a veal's birth is needed to initiate a lactation, and dairy cows are culled at the end of their productive life. It is possible to estimate the mean quantity of meat co-produced per kg of milk produced. We considered 4 lactations for the productive life of a cow (1) producing 6990 kg of milk per lactation (2). Two male and two female as offspring were considered, leading to 1.8 veal calves, i.e. 0.6 steers, 1.5 cows for herd renewal and 0.1 heifers (3). Using estimates of carcass weights (1,3,4), offspring plus culled cow over a productive year represent 823 kg carcass-equivalent, or 20 kg of marketable meat for 1000 L of milk. We considered a 30% weight loss during meat cooking (5), leading to a constraint expressed as *bovine dairy protein (g) ≤ 0.43*bovine meat (g).*

Blood sausage and pork meat

Blood sausage is a deli meat specialty prepared with pork blood. Its high content in heme iron (the readily absorbed form of iron) makes it a potentially valuable food choice to fulfill iron requirements when iron bioavailability is taken into account. As blood being a co-product of pork meat, we constrained blood sausage quantity. We estimated that a hog provides 47.6 kg of cooked meat and 3 liters of blood (6). As it takes 4600 g of blood to produce 9600 g of blood sausage, the constraint was set as *blood sausage (g) ≤ 0.13*pork meat (g).*

References

1. Normabev. Evolutions hebdomadaires des âges et poids des vaches laitières abattues d’après remontées d’abattage (Weekly evolution of age and weight of dairy cows at slaughtering). 2016.
2. Institut de l’élevage, Confédération nationale de l’élevage. Chiffres clés 2015 : Productions bovines lait et viande (2015 key figures. Bovine production milk and meat). 2015.
3. FranceAgriMer. Observatoire de la formation des prix et des marges des produits alimentaires (Observatory of pricing and margins of food products). 2015.
4. FranceAgriMer. Note de conjoncture, Viandes rouges/Filière bovine (Sectoral report, Red meat/Bovine sector). 2015.
5. Duchène C, Gandemer G. Valeurs nutritionnelles des viandes cuites. Effets de la cuisson sur la composition des viandes (Nutritional values of cooked meats. Cooking impact on meat composition). 2015.
6. FranceAgriMer. Étude sur la valorisation du 5e quartier des filières bovine, ovine et porcine en France (Study on repurposing the fifth quarter in the bovine, ovine and pork sector in France). 2013.
